# Model estimates of the burden of outpatient visits attributable to influenza in the United States

**DOI:** 10.1186/s12879-016-1939-7

**Published:** 2016-11-07

**Authors:** Gonçalo Matias, François Haguinet, Roger L. Lustig, Laurel Edelman, Gerardo Chowell, Robert J. Taylor

**Affiliations:** 1 GSK Vaccines, Avenue Fleming 20, Parc de la Noire Epine, Wavre, Belgium; 2 Sage Analytica, 4915 St. Elmo Ave., Suite 205, Bethesda, MD 20814 USA; 3 Symphony Health Solutions, Suite 100, 550 Blair Mill Road, Horsham, PA 19044 USA; 4 Present address: Independent Outcomes and Healthcare Researcher, 1591 White Chimney Road, West Chester, PA 19380 USA; 5 School of Public Health, Georgia State University, Atlanta, GA USA

**Keywords:** General practice, Influenza, Burden of disease, Mathematical model

## Abstract

**Background:**

Although many studies have modelled the national burdens of hospitalizations and deaths due to influenza, few studies have considered the outpatient burden. To fill this gap for the United States (US), we applied traditional statistical modelling approaches to time series derived from large medical claims databases held in the private sector.

**Methods:**

We accessed ICD-9-coded office visit data extracted from Truven Health Analytics’ MarketScan Commercial database covering about one third of the US population <65 years during 2001–2009, and Medicare Supplemental data covering about one fifth of US seniors 65+ during 2006–2009. We extracted weekly time series of visits due to respiratory diagnoses, otitis media (OM), and urinary tract infections (UTI), a “negative control”. We used multiple linear regression modelling to estimate age-specific influenza-related excess in office visits.

**Results:**

In the <65 year age group, in the 8 pre-pandemic seasons studied and for the broadest defined respiratory outcome, the model attributed an average of ~14.5 M (Standard deviation [SD] across seasons 3.9 million) office visits to influenza (rate of 5,581/100,000 population). Of these, ~80 % of visits occurred in the 5–17 and 18–49 age group. In school children aged 5–17 year olds and adult 18–64 year age groups the majority of visits were due to influenza B, while A/H3N2 explained most visits in children <5 year olds. The model further attributed ~2.2 M OM visits (SD across seasons 790,000) annually to influenza, of which 86 % of these occurred in children <18 years; this indicates that 6.4 % of all infants <2 years and 4.9 % of all toddlers aged 2–4 years in the US have an influenza-attributable outpatient visit with an OM diagnosis. In seniors 65 years and older, our model attributed ~0.7 M (SD across seasons 351,000) respiratory visits to influenza (rate of 1,887/100,000 population). The model identified no significant excess UTI (negative control) visits in most seasons.

**Conclusions:**

This is to our knowledge a first study of the outpatient burden of influenza in the US in a large database. The model estimated that 10 % of all children <18 years and 4 % of the entire population <65 years seek outpatient care for respiratory illness attributable to influenza annually.

**Trial registration:**

ClinicalTrial.gov, NCT02019732.

## Background

The true burden of influenza is difficult to measure because many infections cause influenza-like-illness and so laboratory testing is needed to confirm influenza, testing of patients is usually at the discretion of the physician so that many influenza infections will not be recorded in clinical databases, and the clinical manifestations of influenza vary in adults and children, which means that influenza may not be suspected [1, 2]. Because of these difficulties, epidemiologists use indirect statistical approaches to attribute to influenza a portion of seasonal increases in adverse health outcomes that occur during influenza epidemics. These indirect statistical approaches have often been used to estimate the burden of mortality and hospitalisation [3–6]. However, few studies have attempted to quantify the influenza burden in the primary care setting [6–8].

Influenza causes a range of clinical symptoms that are not confined to the respiratory tract, such as gastrointestinal symptoms and febrile convulsions in young children, and sometimes the clinical illness is not particularly severe [9]. For these reasons, influenza is often not properly diagnosed. Otitis media (OM) often prompts young children to consult a physician, and is considered to be a complication of influenza infection [2, 10]. In a recent evaluation of the influenza primary healthcare burden in the United Kingdom (UK), indirect modelling suggested that OM attributable to influenza occurred in children at a similar rate to influenza-like-illness, suggesting that rather than being a complication of influenza, OM is part of the clinical symptomatology of influenza in children [8].

The outpatient burden is important because it represents a substantial portion of the economic healthcare burden caused by influenza; that burden also includes absenteeism due to illness among both adults and their children, so that the full burden of paediatric influenza extends beyond children themselves. Estimates of the influenza burden that are limited to respiratory outcomes, such as influenza-like-illness or acute respiratory infection, will not capture the contribution of non-respiratory diseases causes by influenza, such as OM in children. We used retrospective data extracted from MarketScan databases in a time series model to estimate the burden of influenza-attributable physician office visits in the United States (US) by age, influenza strain and season (www.clinicaltrials.gov NCT02019732). We report on a new outcome, ‘respiratory disease broadly defined’, that combined all respiratory diagnoses with selected presenting symptoms, which was designed to have high sensitivity while maintaining specificity compared to other commonly used outcome definitions such as ‘all-cause’ or ‘respiratory’.

## Methods

### Study design

Multiple linear regression models were used to quantify the burden of multiple mild outcomes (i.e., those that result in visits to a physician’s office) attributable to influenza in the US, stratifying by age and controlling for the contribution of respiratory syncytial virus (RSV).

We included all physician office visits for a) persons <65 years of age with an outcome of interest recorded in the MarketScan Commercial database in the period from July 2001 through March 2009 (eight seasons), and b) persons 65+ years of age with an outcome of interest that were recorded in the MarketScan Medicare Supplemental database in the period from July 2006 through March 2009 (three seasons). Data from individual subjects were not linked over time.

The study was conducted according to International Society for Pharmacoepidemiology Guidelines for Good Pharmacoepidemiology Practices, local regulations and privacy laws. Consent was not needed as all patients were anonymised and only aggregate data are reported here.

### Data sources

The MarketScan databases used in this study are maintained by Truven Health Analytics (formerly Thomson Medstat), and contain claims data from a variety of health plans [11]. The gender, age, and geographic distribution of the MarketScan population can be weighted to create nationally representative samples of Americans covered by health insurance. At the time of the study the databases covered 110.1 million persons <65 years of age and 6.4 million persons aged 65+ years. It should be noted that the two datasets were different and any comparison of the two sets of estimates should be made cautiously.

Weekly influenza surveillance data were obtained from the Centers for Disease Control and Prevention (CDC) [12]. Influenza virology data were collected for weeks 40 of the first year in a season through week 20 in the second year, by approximately 80 US World Health Organization collaborating laboratories and 60 National Respiratory and Enteric Virus Surveillance Systems (NREVSS) laboratories. Weekly RSV surveillance data were obtained from the NREVSS [13].

### Data preparation

We defined several outcomes of varying sensitivity and specificity using International Classification of Disease (ICD)-9 codes (Table 1). We developed an outcome category ‘respiratory disease broadly defined’ that included diseases of the respiratory system as well as fever, cough, abnormalities of breathing and unspecified viral infections. A negative control outcome (urinary tract infection) was used to assess the presence of trends not associated with influenza.

**Table 1:** Outcomes of interest

Description	ICD-9-CM
Respiratory Disease Broadly Defined: Respiratory diseases, fever, cough, abnormalities of breathing, viral infections NOS	460–519, 780.6, 786.1–786.4, 786.7–786.9, 079
Respiratory Disease	460–519
Cardio–respiratory disease	390–519
Otitis media	381–382
Urinary Tract Infection	599.0

For each outcome we extracted weekly time series, stratified by age group and region, of the total number of physician office visits recorded during the week divided by the total of covered persons during the same week; records with any mention of defined codes among the recorded diagnoses were included. Weighting factors based on regional populations of age groups were used to remove substantial year-to-year changes in covered populations that occurred each January.

The influenza virology time series were given by the total number of positives by type and subtype, divided by the seasonal total number of influenza tests. The RSV virology time series were given by the number of positives divided by the seasonal total number of RSV tests. The time series for each outcome were stratified by age group. These were merged into the database of weekly outcome time series, by week and year, and employed as explanatory variables.

### Statistical methods

Each age group and region outcome series was modelled by multiple regression. Numbers of influenza-attributable outcome cases were summed across regions, and converted to national rates. Seasonal 95 % confidence intervals (CI) were computed based on the standard error of the multiple regression model parameter for each individual pathogen. We calculated the weekly point estimates as the product of the regression coefficient and the weekly viral circulation, then aggregated the weekly estimates over the entire season. We repeated the procedure using the lower and upper 95 % estimates for the regression parameter to obtain the seasonal upper and lower CIs.

The variability of seasonal all-age estimates was assessed using standard deviations (SD). This SD represents the variability of the attributable burden between seasons and not the uncertainty of the individual seasonal estimates.

The best model fit for both databases adjusted for major holidays using a dummy variable approach. Cyclic terms (sine and cosine with annual period) and lagging of the virology series were considered but not used in the best-fit models. The use of sine and cosine terms to model seasonal variations in disease incidence and disease burden is pervasive in the literature over many decades, made necessary by the absence of data to model seasonal variation in incidence rates. It is intended to capture the effect of “other pathogens” and “other factors” such as weather. The use of sine and cosine terms is intended to capture the effect of “other pathogens” and “other factors” such as weather in seasonal variations in disease incidence and disease burden. In our initial model, cyclic terms captured a high fraction of the attribution rates across defined outcomes. As a result, the attribution rates assigned to other terms in the model (RSV and influenza virology indicators) tended to be much smaller (and often negative) than attribution rates captured by the cyclic terms. On removing cyclic terms from the model, negative attributions were dramatically diminished.

The final model form for the MarketScan Commercial Database was:

$$ Y={\beta}_1+{\beta}_2*(RSV)+{\beta}_3*\left(A/H1N1\right)+{\beta}_4*\left(A/H3N2\right)+{\beta}_5*(B)+{\beta}_6t+{\beta}_7{t}^2+{\beta}_8{t}^3 $$

where t is the week number, Y is the incidence of an outcome in each time period t, RSV & influenza (A/H1N1, A/H3N2 and B) are the proportions of laboratory isolates during t.

Medicare Supplemental data were limited to three seasons. Collinearity amongst the various virus terms resulted in unstable results and poor fits. We therefore aggregated the influenza terms (A/H1N1, A/H3N2 and B) into a single influenza term in the model; we then made seasonal attributions to each type/subtype according to the seasonal proportion of positive samples. The final model form was:

$$ Y = {\upbeta}_1+{\upbeta}_2*(RSV) + {\upbeta}_3*\left( Aggregated\  Influenza\right) + {\upbeta}_4t + {\upbeta}_5{t}^2+{\upbeta}_6{t}^3 $$

Statistical analyses were performed using SAS 9.3.

## Results

### Model fit

**Fig. 1:**
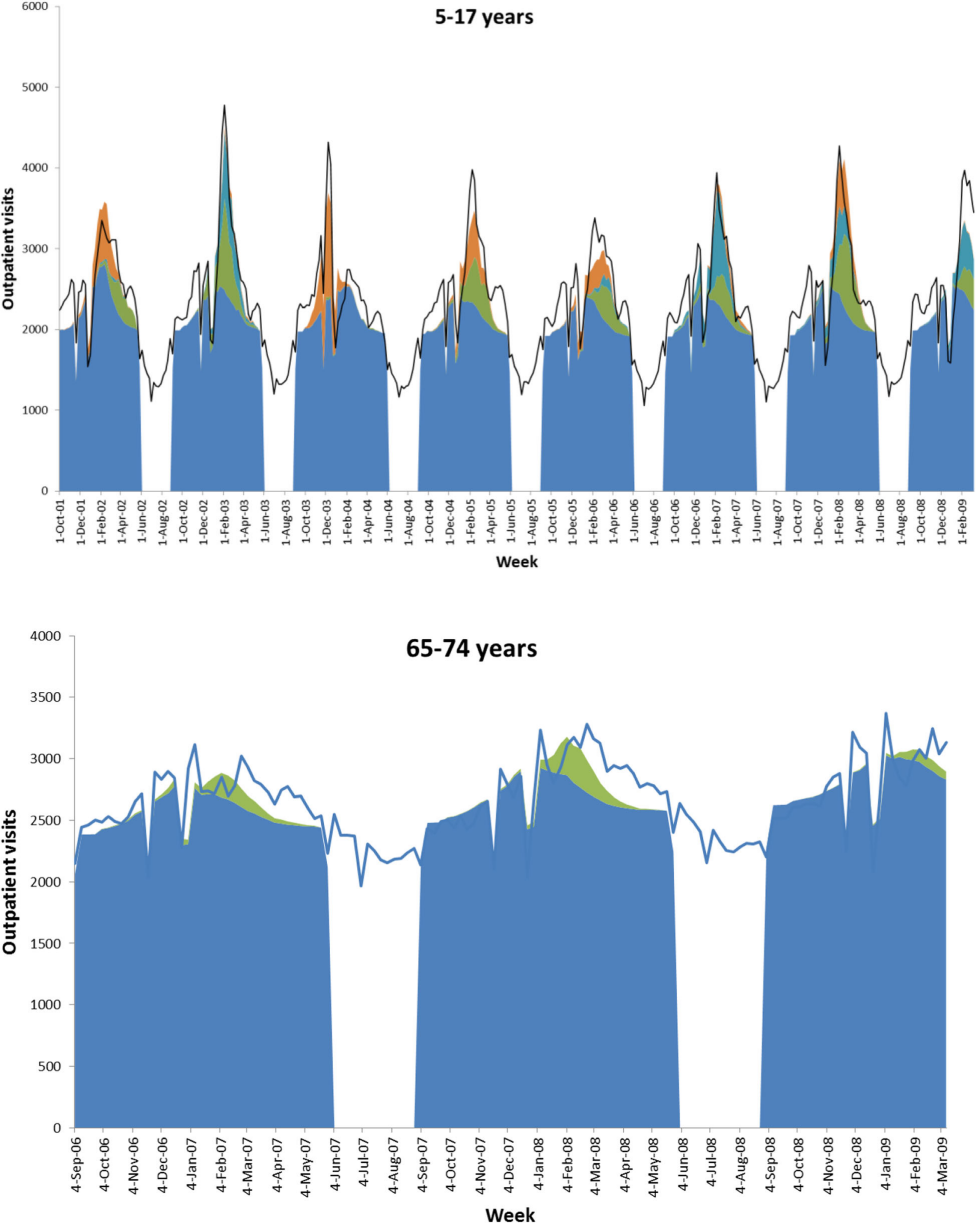
Attribution modelling showing excess office visits attributable to influenza A/H1N1 (*purple*), A/H3N2 (*orange*) and B (*green*) in 5–17 year olds (MarketScan Commercial Database), and influenza of all types (*green*) in 65–74 year olds (MarketScan Medicare Supplemental Database). The sum of the secular trend and RSV attribution is shown in *blue*; the *solid lines* represent the observed number of respiratory (broadly defined) visits

The model fit (R^2^) for physician visits for respiratory disease (broadly defined) ranged from 0.54 to 0.79 in the MarketScan Commercial dataset, and from 0.55 to 0.80 in the Medicare Supplemental dataset. The addition of the virology terms to the model increased R^2^ in all strata, by amounts ranging from 0.29 to 0.70. Figure 1 shows example attributions and model fit to the data.

Between 2001 and 2009, there was an average of 150 million physician visits per season for a respiratory illness (broadly defined) by individuals aged <65 year olds in the US. Of these, an estimated 14.5 million visits (9.6 %) (SD across seasons 3.9 million) were attributable to influenza (Table 2); of which 1.7 % were attributed to influenza A/H1N1, 3.9 % to influenza A/H3N2 and 4.1 % to influenza B. Assuming one office visit per individual, the data correspond to an average of 5.6 primary care visits for respiratory disease per 100 persons aged <65 years annually.

**Table 2:** Estimated counts of influenza-attributable office visits for respiratory (broadly defined), respiratory and cardiorespiratory causes in the United States, 2001–02 through 2008–09

	Respiratory (broad)		Respiratory		Cardiorespiratory	
Season	Estimate	(95 % CI)	Estimate	(95 % CI)	Estimate	(95 % CI)
MarketScan Commercial Database, <65 year olds						
2001–02	12,365,184	(11,665,801–13,064,541)	10,475,575	(9,816,954–11,134,170)	10,194,445	(9,522,888–10,866,003)
2002–03	16,803,325	(15,883,426−17,723,224)	14,701,362	(13,836,458–15,566,240)	14,554,184	(13,664,649–15,443,744)
2003–04	13,618,103	(12,767,860–14,468,321)	11,354,261	(10,556,090–12,152,431)	11,258,742	(10,426,957–12,090,553)
2004–05	15,814,272	(15,089,234–16,539,284)	13,605,842	(12,927,419–14,284,264)	13,643,186	(12,922,048–14,364,325)
2005–06	11,795,968	(11,243,720–12,348,242)	10,071,294	(9,555,005–10,587,610)	10,213,174	(9,660,795–10,765,500)
2006–07	14,628,435	(13,601,384–15,655,487)	12,634,875	(11,674,057–13,595,718)	12,989,309	(11,977,996–14,000,621)
2007–08	21,896,301	(20,932,203–22,860,399)	18,921,369	(18,020,955–19,821,782)	19,535,453	(18,554,039–20,516,840)
2008–09	8,683,598	(8,167,552–9,199,670)	7,535,656	(7,053,640–8,017,619)	7,780,771	(7,262,485–8,299,004)
* Average*	*14,450,648*	*−*	*12,412,529*	*−*	*12,521,158*	*−*
* Std dev*	*3,931,783*	*−*	*3,447,860*	*−*	*3,578,048*	*−*
MarketScan Medicare Supplemental Database, 65+ year olds						
2006–07	708,335	(354,590–1,062,080)	615,166	(286,572–943,759)	265,729	(0–682,663)
2007–08	1,078,071	(535,820–1,620,322)	936,707	(432,929–1,440,484)	396,427	(0–1,026,793)
2008–09	376,923	(185,672–568,173)	327,184	(149,709–504,659)	132,283	(0–343,834)
* Average*	*721,110*	*−*	*626,352*	*−*	*264,813*	*−*
* Std dev*	*350,749*	*−*	*304,915*	*−*	*132,074*	*−*

<65 years olds: 2001–02 through 2008–09 seasons, MarketScan Commercial Database

65+ year olds: 2006–09 seasons, MarketScan Medicare Supplemental Database

Std dev = standard deviation, average = mean seasonal estimate

95 % CI = 95 % confidence intervals

The seasonal average rate of influenza-attributable office visits was highest among children in age groups <18 years (Table 3). Influenza A disease appeared to be less common after age 17 years and decreased further with age. This trend was less marked for influenza B; with high rates of office visits attributable to influenza B respiratory disease persisting until 50–64 years of age.

**Table 3:** Average seasonal model attributions (number and rate per 100,000 population) by influenza sub-type and age

	Influenza A/H1N1				Influenza A/H3N2				Influenza B				All influenza			
Age group	Rate	SD	N	SD	Rate	SD	N	SD	Rate	SD	N	SD	Rate	SD	N	SD
Respiratory broad																
0–1	1148	1292	92,543	104,232	4189	3220	334,583	255,510	1958	1263	157,391	101,799	7295	1933	584,517	155,296
2–4	2163	2353	257,345	279,828	5252	4017	619,022	470,595	2256	1473	267,541	173,631	9672	2312	1,143,907	273,448
5–17	2824	3010	1,517,946	1,618,530	3573	2771	1,914,587	1,481,878	3996	2592	2,145,385	1,391,299	10392	3093	5,577,918	1,667,267
18–49	438	468	590,623	630,727	1787	1399	2,402,020	1,875,906	1979	1282	2,666,447	1,727,718	4205	1274	5,659,089	1,720,149
50–64	55	60	28,749	31,093	1229	957	608,283	458,111	1649	1072	848,186	556,510	2933	1031	1,485,217	555,566
0–64	953	1020	2,487,205	2,658,483	2285	1781	5,878,493	4,539,150	2343	1519	6,084,951	3,940,675	5581	1496	14,450,648	3,931,783
65–74	654	334	130,668	62,250	596	722	119,328	145,317	424	189	85,381	37,417	1674	837	335,377	164,184
75+	811	383	147,352	68,341	757	920	137,573	167,557	574	269	104,445	48,993	2142	1040	389,370	188,526
65+	725	356	276,803	129,745	669	811	255,580	310,995	492	226	188,727	85,855	1887	929	721,110	350,749
Otitis media																
0–1	976	1069	78,683	86,175	1594	1301	127,280	103,167	3884	2515	312,139	202,623	6455	2291	518,102	186,416
2–4	821	859	97,574	102,047	1492	1182	175,651	138,078	2627	1697	311,427	200,700	4939	1613	584,652	191,428
5–17	336	354	180,606	190,499	389	309	208,399	165,421	771	497	414,081	267,052	1496	547	803,086	294,139
18–49	32	34	42,940	45,757	48	37	64,380	50,103	111	72	149,756	97,400	191	74	257,076	100,433
50–64	10	11	5377	5685	18	14	8859	6695	55	36	28,401	18,981	83	37	42,637	20,350
0–64	155	164	405,177	428,584	227	182	584,566	462,874	468	303	1,215,805	785,121	851	301	2,205,547	787,106
65–74	6	2	1267	363	6	6	1102	1231	4	2	826	404	16	7	3195	1447
75+	1	0	140	89	1	1	132	197	0	0	87	21	2	1	359	179
65+	3	1	1196	520	3	3	1069	1336	2	1	730	271	8	4	2994	1437

<65 years olds: 2001–02 through 2008–09 seasons, MarketScan Commercial Database

65+ year olds: 2006–09 seasons, MarketScan Medicare Supplemental Database

*SD* standard deviation of the variability of the attributable burden between seasons, *N* number

Among 65+ year olds, in an average season there were 31,644,400 office visits for a respiratory illness (broadly defined), of which 2.3 % (*n* = 721,110) (SD across seasons 350,749) were for respiratory disease attributable to influenza (Table 2). While the estimated rates of influenza-attributable office visits for respiratory disease were lower in the elderly than in children, the number of office visits in 65+ year olds exceeded those in infants 0–1 year of age (Table 3). The estimated rate of influenza-attributable office visits for respiratory disease was 28 % higher in 75+ year olds than in 65–74 year olds (Table 3).

There was marked inter-seasonal variability in influenza attributions for respiratory (broadly defined) office visits overall, and in the predominant influenza type (Table 2). The highest estimate of influenza-attributable office visits in both databases was in 2007/08, and the lowest estimate was in 2008/09). In these seasons, influenza-attributable respiratory infection (broadly defined) represented 13 and 6 % of the burden of respiratory illness respectively among 0–64 year olds.

Respiratory illness due to influenza in people <65 years was predominantly attributable to influenza A/H3N2 in the 2001/02, 2003/04 and 2005/06 seasons, approximately equally attributable to A/H3N2 and type B lineages in the 2004/05 season, and predominately to type B in the 2002/03, 2007/08 and 2008/09 seasons. Influenza A/H1N1 dominated only in 2006/07, a season in which influenza B also co-circulated (Table 4). The majority of influenza respiratory disease in adults aged 65+ years was attributable to influenza A, with A/H1N1 and A/H3N2 contributing equally (Table 3).

**Table 4:** Estimated counts of influenza-attributable office visits for respiratory disease (broad) in the United States by influenza subtype

	Influenza A/H1N1		Influenza A/H3N2		Influenza B	
Season	Estimate	(95 % CI)	Estimate	(95 % CI)	Estimate	(95 % CI)
MarketScan Commercial Database, <65 year olds						
2001–02	244,346	(206,437–282,230)	9,591,775	(8,949,669–10,233,881)	2,529,062	(2,349,650–2,708,475)
2002–03	4,927,892	(4,226,578–5,629,206)	956,554	(873,630–1,039,452)	10,918,879	(10,010,801–11,826,957)
2003–04	2253	(1715–2791)	13,208,713	(12,356,831–14,060,570)	407,137	(377,772–436,527)
2004–05	26,162	(21,539–30,785)	7,662,099	(7,143,927–8,180,245)	8,126,011	(7,568,687–8,683,335)
2005–06	679,718	(551,518–807,891)	6,668,041	(6,246,233–7,089,875)	4,448,210	(4,124,428–4,771,991)
2006–07	7,070,007	(5,985,195–8,154,818)	1,809,498	(1,686,185–1,932,810)	5,748,931	(5,289,075–6,208,788)
2007–08	3,334,887	(2,864,430–3,805,317)	6,838,330	(6,349,764–7,326,895)	11,723,085	(10,930,663–12,515,507)
2008–09	3,612,374	(3,071,846–4,152,875)	292,933	(273,464–312,428)	4,778,291	(4,434,527–5,122,082)
* Average*	2,487,205	−	5,878,493	−	6,084,951	−
* SD*	2,658,483	−	4,539,150	−	3,940,675	−

<65 years olds: 2001–02 through 2008–09 seasons, MarketScan Commercial Database

95 % CI = 95 % confidence intervals, SD = standard deviation of the variability of the attributable burden between seasons, average = mean seasonal estimate

The largest numbers of influenza-attributable office visits was among school-age children (5–17 year olds) and young adults (18–49 year olds), each accounting for nearly 40 % (or 5.6 million consultations in an average season, SD across seasons 1.7 million) of the medically attended burden of influenza in individuals <65 year olds in the US (Table 3). Overall, 38 and 47 % of all influenza-associated office visits for respiratory disease in these respective age groups were attributable to influenza B. The other influenza subtypes shared a lower but still comparable burden, with 27 and 34 % of the influenza-associated office visits for respiratory disease by children 5–17 year olds attributable to influenza A/H1N1 and A/H3N2, respectively.

**Fig. 2:**
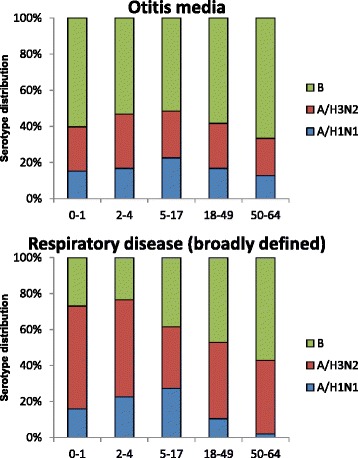
The percentage of the estimated burdens for respiratory and otitis media outcomes attributed to influenza A and influenza B by age group (mean across seasons, MarketScan Commercial database). Footnote: The total number of office visits for each outcome was: Respiratory disease (broadly defined) *N* = 115,605,186, Otitis media *N* = 17,644,379

In an average season we estimate that there were 2,205,547 office visits for influenza-attributable otitis media, representing 10 % of all office visits for OM in <65 year olds in the US. The highest rates of influenza-attributable OM were in children (0–1 and 2–4 year olds) with 50 % of all office visits for OM occurring in these age groups combined (Table 3). There was more OM attributed to influenza B than A in all age groups (Fig. 2). As expected, the burden of visits for OM was low in adults.

### Control outcome

The burden of medically-attended urinary-tract infections attributed to influenza by the model was negligible.

## Discussion

To the best of our knowledge, our study is among the first to estimate the burden of outpatient visits attributable to influenza in the US. Using a novel outcome (respiratory disease broadly defined), the model attributed seasonal means of 14,450,648 (SD 3,931,783) (<65 year olds) and 721,110 (SD 350,749) (65+ year olds) office visits to influenza for respiratory disease.

Children (0 to <18 years of age) have the highest rates of medically-attended influenza as determined by the model. This is consistent with previous studies showing that children are responsible for most influenza-attributed doctor visits in the US and UK [6, 8, 14], but contrasts with results of Molinari et al. [15] who used a probabilistic model and estimated 31.4 million outpatient visits for influenza among all age groups in 2003, with the highest number of consultations in 65+ year olds. The discrepancy between these results compared to our own and other studies probably reflects the lack of precision associated with the probabilistic approach, which relied on assumptions for influenza consultation rates guided by published clinical trials or epidemiological studies.

While young children and adolescents have the highest rates of office visits for influenza attributable respiratory infections, some other studies indicate that the elderly exhibit the highest influenza-attributable hospitalisations and mortality rates (Table 5).

**Table 5:** Seasonal Influenza attributions (rate per 100,000 population) in selected studies conducted in the US and Europe

Country	Study year	Data source	Outcome	Age (yrs)	Event rate (average per season/100,000 population)
UK [8]	1995–2009	Clinical Practice Research Datalink	Respiratory disease (broadly defined) general practitioner visits	0–4	2975
				5–17	2213
				18–49	1187
				50–64	1255
				65+	1216
				All	1460
European Paediatric Influenza Analysis project [17]	UK, 2002–2008	RCGP Weekly Returns Service	Influenza-like-illness outpatient visits	0–4	384
				5–17	315
	Italy, 2003–2008	surveillance network		0–4	9229
				5–17	8915
	Netherlands 2002–2008	surveillance network		0–4	925
				5–17	121
	Spain, 2002–2008	Spanish Influenza Sentinel Surveillance System		0–4	2,156
				5–17	2,955
US [16]	2001–2006	Electronic reporting of emergency department chief complaint data (New York city)	“fever and respiratory” syndrome	<2	540–7710
				2–4	610–4790
				5–12	380–1230
				13–17	140–820
				18–39	80–440
				40–64	60–340
				65+	50–570
US [24]	1993–2008	The Healthcare Cost and Utilization Project	Influenza-hospitalizations	<1	151/100,000 p–y
				1–4	39/100,000 p–y
				5–49	17/100,000 p–y
				50–64	6/100,000 p–y
				65+	309/100,000 p–y
				All	64/100,000 p–y
US [25]	1997–2007	National Center for Health Statistics	All-cause deaths	All	11.92
			Respiratory deaths	All	3.58
US [3]	1997–2009	US National Vital Statistics System	Respiratory disease (broadly defined) deaths	0–4	0.5
				5–17	0.2
				18–49	0.6
				51–64	3.5
				65–74	14.1
				75+	80.1
				All	6.6

*RCGP* royal college of general practitioners, *p–y* person years

Olson et al. [16] reported excess visits for “respiratory and fever” syndrome in emergency departments in New York City between 2001 and 2006. The burden of influenza among children <2 and 2–4 years of age estimated in the present study was higher than the range determined by Olson et al. for comparable years (range 6508–9633 visits per 100,000 population versus 540–7710 visits per 100,000 population in <2 year olds, and 8226–11,959 versus 610–4790 per 100,000 population in 2–4 year olds, respectively), and was higher for other comparable age groups. This difference might be expected if emergency department visits represent only a fraction of office visits. In another study, Fowlkes et al. [14] used 38 outpatient practices to estimate the incidence of medically attended influenza at the community level. Overall, our estimates were in reasonable agreement with those published by Fowlkes et al. However, that study was restricted to a single season (2009/10), in which the influenza A/H1N1 pandemic strain vastly predominated in the US.

Two studies set in the UK assessed the burden of influenza-attributable respiratory illness in primary care. Our estimates are higher than those of Pitman et al. [6] and Fleming et al. [8] for all comparable age groups. The estimates of the burden of medically-attended influenza produced by Pitman were restricted to a single season (2002/03), which was a mixed A/H3N2 and B season. Burden estimates produced using a single season can be unstable and may be affected by a number of factors, including random inter-seasonal variations in the number of cases diagnosed with a respiratory disease as well as changes in the type and severity of the predominant circulating influenza strain. Wide disparity in the attributable influenza burden between European countries was also noted by Paget et al. [17], who reported seasonal averages for influenza-attributable influenza-like-illness of between 0.4 and 18 % in individual countries (Table 5). In view of the dissimilarities between individual countries, which include differences in population, viral circulation patterns, climatic conditions, healthcare systems and patterns of healthcare utilisation (especially costs and incentives for seeking care), close agreement between studies conducted in the UK and the present estimates would not be anticipated.

The model attributed a large number of visits for OM to influenza. As expected, the majority of the influenza-attributable OM burden was in younger age groups. We estimated that 10 % of all office visits for otitis media in <65 year olds were attributable to influenza, and that 6 % of all 0–1 year olds attend for an influenza-attributable otitis media office visit in an average season. Among 0–1 year olds, the seasonal rate of office visits for influenza-attributable OM was close to that of respiratory disease (broadly defined), lending support to the notion that otitis media is part of the primary symptomatology of influenza in this age group, rather than a complication of influenza [8]. Clinical trials have suggested that influenza vaccination reduces OM in children [18–20], although one trial conducted in young children (6–24 months) showed no impact of influenza vaccination on OM or on healthcare utilization, suggesting a potential age-related impact on efficacy [21].

As is typical of influenza, the influenza-attributable burden of physician office visits was highly strain and age specific, and varied by season. The 2003/04 season was characterised by a severe A/H3N2 epidemic, whereas a negligible burden was observed in the immediately preceding season. The relative impact of influenza A/H1N1 was small in some seasons for most age groups, but A/H1N1 particularly affected the young (those <18 year olds) and the elderly (65+ year olds) in 2006/07. Influenza A/H3N2 and B shared most of the burden in adults aged 18–64 years. Among older adults (65+), the attributed burden of respiratory disease was highest for influenza A/H1N1.. Influenza B caused more office visits for respiratory illness than either A/H1N1 or A/H3N2 in individuals between 5 and 64 years of age. Influenza type B also caused more OM than A/H1N1 and A/H3N2 combined in all age groups <65 years.

Unexpectedly, the estimated burden of influenza B among adult 50–64 year olds was higher than the burden of influenza A/H1N1 and A/H3N2. Although possibly a valid finding, this result is at odds with epidemiological expectations from previous reports which have shown a predominantly higher burden associated with influenza A/H3N2 in adults. It is possible that the proportion of the attributed burden of A/H3N2 in adults was lower than expected due to the higher temporal overlap between A/H3N2 and influenza B virology peaks in some seasons, undermining the model’s ability to appropriately allocate burden to influenza A/H3N2 in such cases.

We optimized the ICD9-coded outcome definition for respiratory illness. Our broader-than-usual definition of a respiratory visit, which included all respiratory diagnosis codes plus fever, cough, abnormalities of breathing and unspecified viral infections, was more sensitive than respiratory disease codes alone while retaining good specificity. By contrast, cardio-respiratory visits yielded fewer influenza-associated cases, possibly because this outcome is likely too non-specific, or because individuals with cardiorespiratory illness are more likely to be hospitalized and less likely to receive primary care.

We recognise several potential limitations of this study. The US has no nationwide data available to study the outpatient disease burden. We used a convenience sample (MarketScan databases) which may be subject to unknown biases and which we extrapolated to a national level. We used two different datasets, which means that the two sets of estimates should be compared cautiously. The available time series for the Medicare database was short (three seasons) requiring use of an aggregated influenza term with distribution to each of the influenza types based on the relative proportion of counts for each strain within influenza-positive specimens. This assumed that the temporal pattern of circulation of each influenza type is the same, and these strains are equally likely to cause a case of the disease outcome modelled. Our model also assumed that the collection of viral data and the detection of outcome events was homogeneous from season to season. This means that the proportion of viral circulation captured, and the conditions under which it was captured, remained the same in each season. We excluded cyclic terms from the final model, and in so doing excluded a seasonal baseline designed to control for unspecified seasonal factors associated with increased morbidity. Although similar in shape to the cyclic term, the RSV virology series had a lower amplitude, and for older age groups peaked earlier than the cyclic pattern associated with the respiratory disease outcome series. As a consequence, a larger fraction of the burden that would be otherwise attributed to the cyclic component may have been attributed to influenza. Overall attribution rates in our study were significantly higher across age-groups compared to models that incorporate cyclic terms, in agreement with a prior study that presented results for both model types [6], suggesting that we may have overestimated the influenza burden. In addition to producing larger attribution rates for influenza, the exclusion of the cyclic term increased estimates of the RSV attributable burden. We did not assess autocorrelation, which could influence the quality of the modelling approach. The OM and respiratory illness burdens are not additive, and it is likely that many patients with an OM also had a respiratory diagnosis. Finally, the model predicted increased visits for respiratory disease in autumn in all of the age groups studied. In the absence of virology results for this period, we are unable to identify the cause of these autumnal peaks, although others have identified increases in respiratory virus infections such as rhinovirus and asthma during autumn in the northern hemisphere [22, 23]. Because a direct comparison with equivalent studies is not possible, the generality of the present findings remains to be determined. However, the observation that the present estimates were consistent with expected age-and outcome-specific patterns long-established in the literature gives confidence about these results.

## Conclusion

The respiratory burden of medically-attended influenza illness in the US is high, and is strain- and age-specific, with considerable seasonal variation. Unexpectedly, influenza B was attributed to more influenza-related office visits during the study period than either influenza A/H3N2 or A/H1N1. The highest rates of office visits attributable to influenza were among children. The burden of influenza-attributable OM in young children is similar to that of respiratory disease. This is one of few studies to model statistically the respiratory burden of medically-attended influenza illness in the US, and the contribution of influenza-attributable otitis media to that burden. Whether it is a primary symptom of influenza in children, or a complication of influenza, OM is theoretically preventable by vaccination. Understanding all influenza-related outcomes that contribute to the outpatient burden is important in assessing the cost-benefit of influenza vaccination programmes.

## Abbreviations

CDC: Centers for disease control and prevention
ICD: International classification of disease
NREVSS: National respiratory and enteric virus surveillance systems
RSV: Respiratory syncytial virus
UK: United Kingdom
US: United States
UTI: Urinary tract infection
